# Electromagnetic Shielding Enhancement of Butyl Rubber/Single-Walled Carbon Nanotube Composites via Water-Induced Modification

**DOI:** 10.3390/polym15092101

**Published:** 2023-04-28

**Authors:** Xin Guo, Guangye Liu

**Affiliations:** Engineering Research Center of High-Performance Polymer and Molding Technology, Ministry of Education, Qingdao University of Science and Technology, Qingdao 266042, China; 0018020008@mails.qust.edu.cn

**Keywords:** electromagnetic shielding, water-induced modification, multi-absorbing

## Abstract

Electromagnetic properties of polymer composites strongly depend on the loading amount and the completeness of the filler’s dispersive structure. Improving the compatibility of single-walled carbon nanotubes (SWCNTs) with isobutylene butyl rubber (IIR) is a good solution to mitigate aggregation. The change in configuration of poly-oxyethylene octyl phenol ether (OP-10) was induced using water as the exposed hydrophilic groups linking with water molecules. The SWCNT and IIR/SWCNT composites were then prepared via wetly-melt mixing at a relatively high temperature to remove water, and they were then mixed with other agents after vacuum drying and cured. The SWCNTs were dispersed uniformly to form a good network for a lower percolation threshold of the wave-absorbing property to 2 phr from 8 phr. With 8 phr SWCNTs, the tensile strength of the material improved significantly from 7.1 MPa to 15.1 MPa, and the total electromagnetic shielding effectiveness of the material was enhanced to 23.8 dB, a 3-fold increase compared to the melt-mixed material. It was demonstrated that water-induced modification achieved good dispersion of SWCNTs for electromagnetic shielding enhancement while maintaining a wide damping temperature range from −55 °C to 40 °C with a damping factor over 0.2.

## 1. Introduction

With the wide use of electrical devices, electromagnetic pollution becomes more general and even universal in our lives and workplaces, which could be harmful to the health of humans and cause interference among equipment. Traditional electromagnetic shielding materials filled with metal are too heavy to wear and not flexible enough to satisfy requirements. Rubber is a good option as a flexible and soft electromagnetic shielding material; however, rubber becomes less flexible, and damping is reduced with too much metal filler. Therefore, an excellent damping polymer and a light-weight filler are desired. With a density of 1.3 g/cm^3^, single-walled carbon nanotubes (SWCNTs) are multi-functional fillers for rubber composites for use in dielectric materials [1], electrically conductive materials [2], and electromagnetic interference (EMI) shielding materials [3,4,5,6,7,8,9,10], thus improving the mechanical properties, the thermal conductivity, and the electrical conductivity of materials due to their high moduli and electrical and thermal conductivities [11,12,13]. Isobutylene butyl rubber (IIR) is widely used in rubber products as it exhibits high damping performance and low gas permeability since it contains a great amount of methyl groups on both sides of the main chain [14]. However, the performance of isobutylene isoprene rubber/single-walled carbon nanotubes (IIR/SWCNTs) is not enhanced as designed because the dispersion of SWCNTs with large aspect ratios is not uniform but results in a great deal of physical entanglement and aggregation in the rubber matrix. Several processes and methods have been proposed and studied to solve the entanglement and aggregation problem, such as melt mixing [15], solution mixing [16,17] and latex mixing [18,19]. Carbon nanotubes (CNTs) are dispersed in the rubber matrix through strong shear forces in the melt-mixing process, but carbon nanotubes with a diameter in the range of tens of nanometers are difficult to disperse uniformly through shearing in incompatible rubber matrices [15]. In the solution-mixing process, the rubber and carbon nanotubes are mixed together in a solvent under specified conditions, and the mixed solution is casted to films on a smooth substrate. However, the agglomeration of carbon nanotubes is a serious problem in the rubber matrix during the solvent evaporating period, which affects the performance of the composite [16,17]. In the latex-mixing process, deionized water is used to disperse carbon nanotubes in advance and cast them on glass plates or other smooth surfaces, after mixing them with latex, before they are finally placed in an oven to remove water completely [20]. This process leaves a large number of carbon nanotubes dispersed in the matrix in the form of aggregates due to the polarity difference between carbon nanotubes and rubber.

Melt mixing is the preferred process for rubber due to its shear-sensitive characteristics, and a general open-mill mixer and an internal mixer can usually be used for production on compatibility between rubber and CNTs. The pre-dispersion process and the modification of CNTs have attracted the interest of researchers in order to improve the uniformity of mixing and dispersion, which can solve the aggregation problem of carbon nanotubes to a certain extent. Physical and chemical modification and combined methods are generally applied. High-power mechanical grinding and ball milling of CNTs form lattice defects or lattice distortions to produce highly active free radicals on the surface of carbon nanotubes, thereby improving the reaction between carbon nanotubes and the rubber matrix. However, it is not easy to control during grinding, resulting in shortening the length of carbon nanotubes and compromising the original properties [21]. The chemical introduction of functional groups, such as carboxyl, hydroxyl, and amino groups with activity, can significantly change the chemical reactivity of the surface of carbon nanotubes with concentrated or diluted acid, neither of which are environmentally friendly. However, compatibility is not improved without sacrificing the original advantages of CNTs [22].

IIR/SWCNT composites do not achieve good EMI performance with fewer SWCNTs through melt mixing. Aggregates of single-walled carbon nanotubes are formed in the matrix in the mixing process because of the large aspect ratio of SWCNTs, leading to higher loading and lower performance.

In attempts to solve the aggregation problem in our research, a poly-oxyethylene octyl phenol ether aqueous solution improved dispersion and the tensile strength, and water could be removed completely. This research result encouraged us to apply this process to improve electromagnetic shielding effectiveness for IIR/SWCNT composites.

Compared to the solution and emulsion methods mentioned above, pure water and a small amount of surfactant agent were used without pollution, and the process of preparation can be conducted in a simple and green way that is easy to master. All the equipment used in the process is universal in manufacturing rubber products, resulting in the potential to scale production.

The uniform dispersion of SWCNTs improves their mechanical properties due to good compatibility with rubber and also improves their thermal conductivity, dielectric properties, and electromagnetic interference shielding effectiveness; further, the percolation threshold of electromagnetic properties is relatively low. In order to fix the entanglement problem by improving compatibility with the IIR matrix, a water-induced modification method is provided in this work to enhance the EMI shielding effectiveness of highly damping IIR/SWCNT nanocomposites at a relatively low percolation threshold concentration of wave properties.

## 2. Materials and Methods

### 2.1. Materials

Isobutylene isoprene rubber (with the product name of IIR 268) was obtained from Exxonmobil Corporation, US. Zinc oxide (ZnO, purity of 99.8%), stearic acid (purity of 97%), sulfur (purity of 99.5%), benzothiazole disulfide (accelerator DM, purity of 99%), and tetramethylthiuram disulfide (accelerator TMTD, purity of 99%) were purchased from the Weifang Qinglian Chemical agents Co., Ltd. (Weifang, China) and were of industrial grade. SWCNTs (CNT-100, 1~2 nm in diameter, 30 μm in length, purity of 90%) were provided by Beijing Deke Daojin Co., Ltd. (Beijing, China). Poly-oxyethylene octyl phenol ether (OP-10, hydrophile-lipophile balance value of 14.5) was used as an emulsifying agent from Guangdong Zhongbang Fine Chemical Co., Ltd. (Guangzhou, China). All the materials were applied in this study without further purification. Pure water was made in the lab to induce the configuration change in OP-10 in order to modify the single-walled carbon nanotubes in a good dispersion state first in OP-10 solution and then in IIR matrix.

### 2.2. Preparation of Samples in Traditional Melt-Mixing Process

In this phase of the experiment, 0 phr (per hundred rubber in weight), 2 phr, 4 phr, 6 phr, or 8 phr single-walled carbon nanotubes were mixed in an open two-roll mixing mill of laboratory size (Φ160 mm × 320 mm, Shanghai Shuangyi Rubber & Plastic Co., Ltd., Shanghai, China) at room temperature with 100 phr IIR, 5 phr ZnO, 3 phr stearic acid, 0.5 phr sulphur, 1 phr accelerator DM, and 0.5 phr accelerator TMTD, respectively, and the prepared samples were vulcanized at 170 °C and marked as D-IIR/0SWCNTs (D is the dry state in melt mixing), D-IIR/2SWCNTs, D-IIR/4SWCNTs, D-IIR/6SWCNTs, and D-IIR/8SWCNTs.

### 2.3. Preparation Samples in Wetly-Melt Mixing via Water-Induced Modification of SWCNTs

The surfactant OP-10 aqueous solution was prepared at the concentration of 2 g/L. Following this, 0 phr, 1 phr, 2 phr, 4 phr, 6 phr, and 8 phr single-walled carbon nanotubes were dispersed in 25 g OP-10 solution, respectively, to prepare the wetly-melt mixed samples marked as IIR/0SWCNTs, IIR/1SWCNTs, IIR/2SWCNTs, IIR/4SWCNTs, IIR/6SWCNTs, and IIR/8SWCNTs. These mixtures were vigorously ultrasonicated in a KH100 ultrasonic bath for 30 min at an effective power of 800 W. In the mixing process, IIR was mixed in an open two-roll mixing mill of laboratory size (Φ160 mm × 320 mm, Shanghai Shuangyi Rubber & Plastic Co., Ltd., Shanghai, China) when the mills were heated to 100–110 °C. Following this, the SWCNTs-OP-10 suspension was added to the IIR on the mills after 15 to 20 min to obtain an IIR–SWCNTs blend. After vacuum drying at 50 °C under the vacuum pressure of −0.096 MPa, the IIR and SWCNTs blend was mixed with other chemical agents. The chemical agents included 5 phr ZnO, 3 phr stearic acid, 0.5 phr sulphur, 1 phr accelerator DM and 0.5 phr accelerator TMTD. And a vulcanization process was followed to prepare the final samples at 170 °C.

### 2.4. Test Instruments and Methods

The dispersive network micro-morphology of SWCNTs in IIR matrix was visualized with the transmission electron microscope (TEM) JEM2100 (Electronics Co., Ltd., Kyoto, Japan) after an ultrathin sample slice was obtained using a slicer Ultramicrotome EM UC7 (Leica, Wetzlar, Germany). Vulcanization parameters of the mixed compounds were tested in RPA2000 (Alpha Technology Inc., Bellingham, WA, USA) at 170 °C under a determined pressure, and the vulcanization time was 2 min longer than t_90_ for the vulcanization of 2 mm sheets under the pressure of 10 MPa. Mechanical properties were performed in a uniaxial tensile machine (AI-7000s, GOTECH testing machine corporation, Taiwan, China) at the speed of 500 mm/min. The electric conductivity of composites was measured with a broadband dielectric resistance spectrometer NOVOCONTROL (Beijing Huidexin Technology Co., Ltd., Beijing, China) after being sputtered with gold layers to the flat surfaces on both sides of the sample with the diameter of 25 mm with a small ion-sputtering apparatus SBC-12 (Beijing Zhongke Technology Instrument Co., Ltd., Beijing, China). Magnetic, dielectric, and electromagnetic shielding effectiveness were measured with the vector network scanner R&S ZNB20 with waveguide clips (SCHWARZ, Baden-Württemberg, Germany) from 8000~12,400 MHz via a cuboid specimen of 22.86 mm in length, 10.86 mm in width, and an actual thickness measured with a vernier caliper. The real part ε′ and imaginary part ε″ of the dielectric constant and the real part μ′ and the imaginary part μ″ of the permeability of composites were measured using a vector network scanner, as mentioned above. As the characterization parameter of microwave absorbing performance, the reflection loss was calculated using the Matrix Lab software (Matlab R2022b, The MathWorks, Inc., Alpharetta, GA, USA) corresponding to simulation of matching thickness and frequency of incident wave according to the real part ε′ and imaginary part ε″ of the dielectric constant and the real part μ′ and the imaginary part μ″ of the magnetic permeability. Dynamic mechanical analysis (DMA) was conducted at the frequency of 30 Hz from −80 °C to 40 °C on DMA/SDTA86 (Mettler-Toledo, Zurich, Switzerland).

## 3. Results and Discussion

### 3.1. Dispersion Structure Characterization

**Figure 1:**
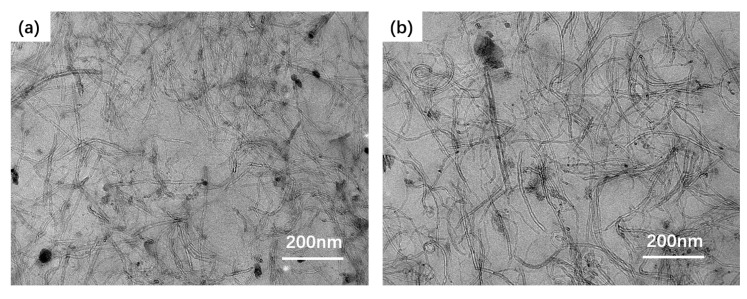
Transmission electron microscope photographs of isobutylene isoprene rubber composites with 8 phr SWCNTs (scale bar is 200 nm). (**a**) Melt-mixed composite. (**b**) Wetly-melt-mixed composite.

The dispersion structure of SWCNTs in the IIR/SWCNT composites with eight SWCNTs via melt mixing and wetly-melt mixing are shown in Figure 1 by visualizing their ultrathin slices in transmission mode. Compared to the partially aggregated state of single-walled carbon nanotubes in D-IIR/8SWCNTs composite produced via melt mixing shown in Figure 1a, single-walled carbon nanotubes are dispersed relatively uniformly and close to the mono-dispersion in the TEM photograph in Figure 1b showing IIR/8SWCNTs treated with wetly-melt mixing. With better dispersion of the single-walled carbon nanotubes in the isobutylene-isoprene rubber matrix, the dispersion network is constructed with fewer defects and aggregations to improve electrical conductivity and mechanical properties for high performance.

**Figure 2:**
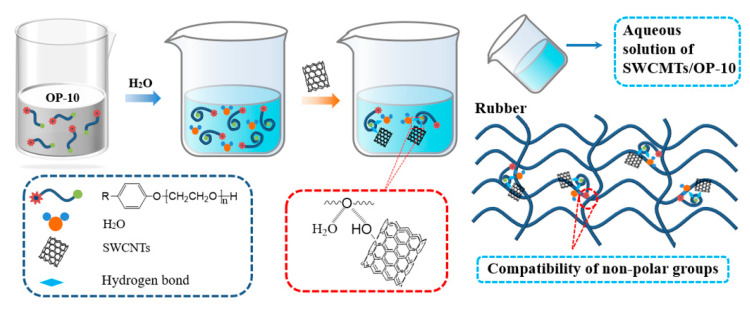
Schematic diagram of dispersion mechanism of single-walled carbon nanotubes in isobutylene isoprene rubber via water-induced modification of SWCNTs.

As shown in Figure 2, the OP-10 chain contains a long nonpolar group useful for its strong hydrophobic ability. In addition, its hydrophilic and lipophilic balances are low, and the hydrophilic group is hidden in the lipophilic group without water. However, the molecular spatial configuration of OP-10 is induced to change under the action of water, forming a tortuous structure when water is mixed with the OP-10 emulsifier. The hydrophilic groups of OP-10 therefore become exposed to water molecules, while the hydrophobic groups are wrapped with the hydrophilic groups. Through the connection of a hydrogen bond with the ether group of OP-10 and water molecules, SWCNTs are very well dispersed in the OP-10 emulsifier aqueous solution.

During the wetly-melt mixing process, the SWCMTs/OP-10 aqueous solution is added to the rubber matrix directly to avoid dry aggregation. Nonpolar groups of OP-10 are then released from being wrapped with the hydrophilic groups. The good compatibility of the nonpolar groups of OP-10 with IIR rubber greatly improves SWCNTs’ dispersion in the rubber matrix.

### 3.2. Vulcanization Properties

**Figure 3:**
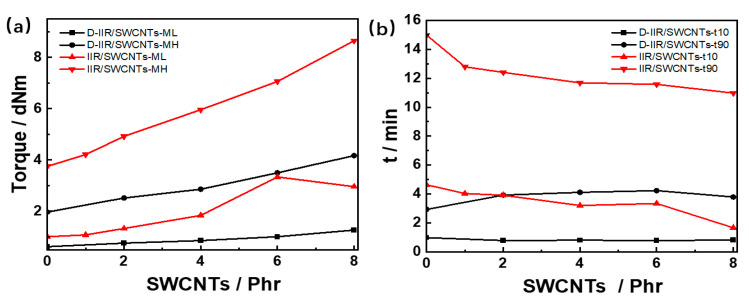
Vulcanization parameters of IIR/SWCNT composites via melt mixing and wetly-melt mixing. (**a**) MH and ML of composites corresponding to the amount of SWCNTs. (**b**) t_10_ and t_90_ of composites corresponding to numbers of SWCNTs. Note: ML—Lowest torque of curing curve; MH—maximum torque of curing curve; MH-ML—the difference between maximum torque and lowest torque, t_10_—the time corresponding to a 10% increase in torque, t_90_—the time corresponding to a 90% increase in torque.

As shown in Figure 3, both ML and MH of isobutylene isoprene rubber composites produced by melt mixing and wetly-melt mixing improved when the amount of SWCNTs increased in the composites, as shown in Figure 3a. There are various polar groups on the surface of SWCNTs as physical crosslinking points to cause greater entanglement between isobutene–isoprene rubber molecular chains, which contributes to the total crosslinking density for higher MH and ML. Single-walled carbon nanotubes are provided more opportunities in a mono-dispersed state to contact with the rubber chains to form more physical crosslinking points, resulting in a much higher MH for IIR/8SWCNTs composites prepared via wetly-melt mixing. Both t_10_ and t_90_ of IIR/SWCNTs show a downward trend with the increasing loading phr of SWCNTs, as shown in Figure 3b. The first reason for this is that the thermal conductivity of single-walled carbon nanotubes at room temperature reaches up to 3500 W/m·K, which is far more than the thermal conductivity of 0.09 W/m·K of butyl rubber at room temperature. The more single-walled carbon nanotubes fill in the compound, the less time it needs for torque increment and vulcanization. The more uniform the dispersion of single-walled carbon nanotubes, the more perfect the heat transfer network is. Additionally, the alkalinity on the surface of SWCNTs accelerates the vulcanization process to perform shorter t_10_ and t_90_.

### 3.3. Mechanical Properties

**Figure 4:**
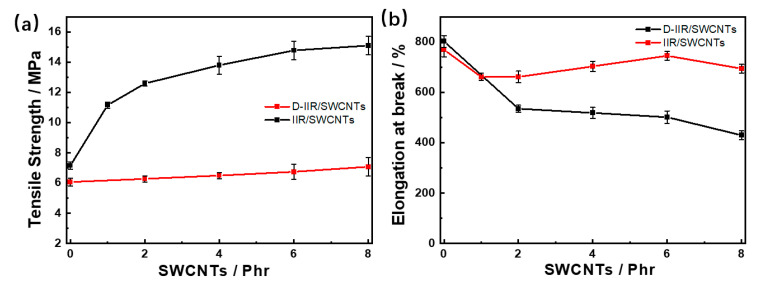
Mechanical properties of IIR/SWCNT composites via melt mixing and wetly-melt mixing. (**a**) Tensile strength corresponding to the loading phr of SWCNTs. (**b**) Elongation at breaking point corresponding to the loading phr of SWCNTs.

Single-walled carbon nanotubes can serve as an excellent reinforcing filler, especially in the uniform dispersive state, because of the high Young’s modulus of 1 TPa [12]. As shown in Figure 4a, the tensile strength of IIR/SWCNT composites positively corresponds to the number of SWCNT_S_. The composite with 8 phr SWCNTs reaches up to 15.09 MPa via wetly-melt mixing, which is 2.1 times that of IIR/8SWCNTs (7.07 MPa) via melt mixing. The elongations at the breaking points of composites prepared via wetly-melt mixing show excellent strain tolerances of over 650%, as shown in Figure 4b. The elongation at the breaking point of the IIR composite without SWCNTs reaches 760%, featuring high elasticity of isobutylene-isoprene rubber. The introduction of a small number of SWCNTs, i.e., less than 2 phr, is not enough to form a network contributing to higher elongation; yet, it causes a discontinuous network and defects in the matrix, resulting in lower elongation. With the addition of more SWCNTs from 2 phr to 6 phr, the elongations of composites increase, which can be attributed to the SWCNT-reinforced network via alkaline promotion of SWCNTs, slidable, entangled, and moveable molecular chains. When the filling of SWCNTs is further increased from 6 to 8 phr, the cross-linking density of the IIR/SWCNT composite increases further, whereas the mobility of molecular chain decreases because of more physically tangled points of SWCNTs. As a result, the elongation is shorter with more than 6 phr SWCNTs in the composite. For the composite materials prepared via melt mixing, the aggregation particles inside the isobutylene isoprene rubber matrix function as a stress concentration point for early failure, and contribute to better elongation, and even more severely when the number of SWCNTs increases.

### 3.4. Dielectric Properties

**Figure 5:**
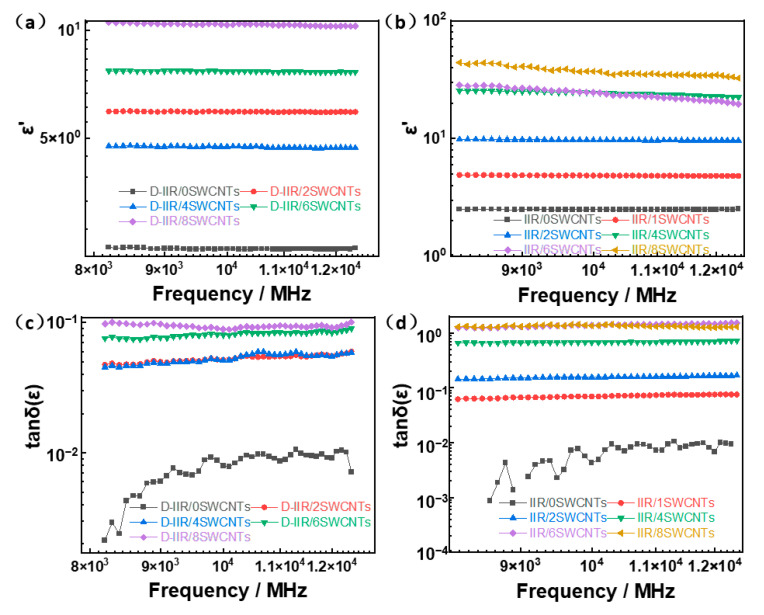
Dielectric properties of IIR/SWCNT composites. (**a**) Dielectric constant (ε′-real part) of melt-mixed composites. (**b**) Dielectric constant (ε′-real part) of wetly-melt-mixed composites. (**c**) Dielectric loss factor of melt-mixed composites. (**d**) Dielectric loss factor of wetly-melt-mixed composites.

As shown in Figure 5a,b, the dielectric properties of isobutylene isoprene rubber composites correspond to the loading phr of single-walled carbon nanotubes from 0 to 8 phr during the frequency range from 10^0^ Hz to 10^7^ Hz. The dielectric constant (ε′-real part) of melt-mixed composites and wetly-melt-mixed composites improve with the number of SWCNTs in the materials, owing to the conductive one-dimensional structure and the insulating butyl rubber, which together form a number of micro-capacitors improving the ability of the composites to store charge.

As the SWCNT content increases, the one-dimensional carbon nanotubes bond to each other in the IIR matrix, gradually building a conductive network. When the SWCNT content increases to a certain critical value, the conductive network can be formed, and the composite material realizes the transformation from an insulator to a semi-conductor, and the conductivity and dielectric constant rise significantly to show “percolation”, as mentioned in our previous work [23]. A high dielectric constant of composite is harmful to impedance matching when used in electromagnetic shielding. It causes many electromagnetic waves to be reflected rather than being absorbed. Usually, it is a semi-conductor material, which affects the conductivity of SWCNTs, so as to reduce the dielectric constant with fewer SWCNTs and improve the effect of impedance matching. The dielectric loss factor of melt-mixed composites and wetly-melt-mixed composites are shown in Figure 5c,d. The dielectric loss of wetly-melt-mixed material is also relatively high at 1.43, while it is 0.1 for melt-mixed material when both are filled with 8 phr SWCNTs. An interconnecting network forms, as shown in TEM images, and a dielectric constant of the IIR /SWCNT composite shows a low percolation threshold value due to the high aspect ratio of SWCNTs and better dispersion. The main reason for this is that good modification dispersion induced by water in OP-10 aqueous solution provides more interface between the butyl rubber molecular chains and SWCNTs for more interfacial polarization with charge accumulation at the interfaces. It is also demonstrated that the influence of loading concentration on the dielectric property lessened when the loading phr was greater than 4.

### 3.5. Electric Conductivity

**Figure 6:**
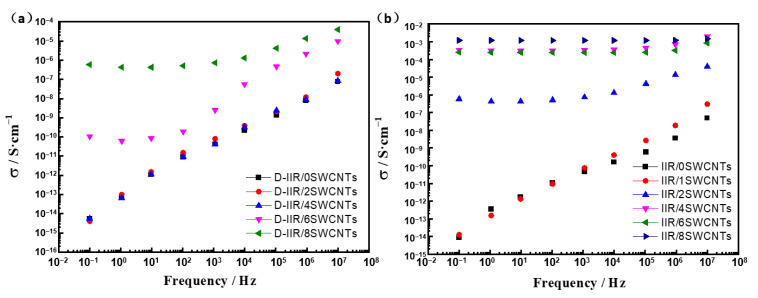
Electric conductivity of IIR/SWCNT composites. (**a**) Melt-mixed composites. (**b**) Wetly-melt-mixed composites.

The electric conductivity of composites is enhanced with the increasing number of SWCNTs loading, as shown in Figure 6. Via a mixed process, the electric conductivity of the IIR/SWCNT composites achieves a threshold transition at 6 phr of SWCNTs, as shown in Figure 6a, while it shows a lower threshold at 2 phr in Figure 6b when a water-induced modification and wetly-melt-mixed process are applied. At the same number of 8 phr SWCNTs, the wetly-melt-mixed sample features the electric conductivity of 10^−3^ S·cm^−1^ due to more paths for electro flow in a continuous network, which is much higher than the melt-mixed one at 10^−6^ S·cm^−1^.

### 3.6. EMI Shielding Effectiveness

**Figure 7:**
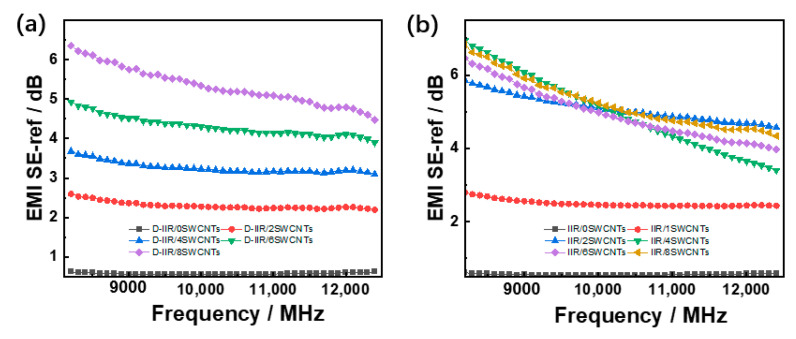

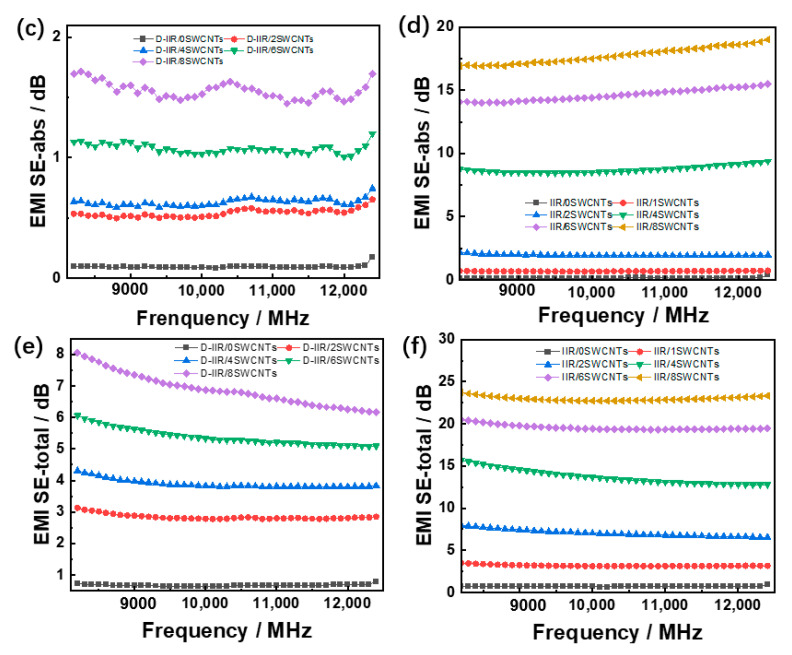
Electromagnetic interference shielding effectiveness of IIR/SWCNT composites. (**a**) Reflection effectiveness of melt-mixed composites. (**b**) Reflection effectiveness of wetly-melt-mixed composites. (**c**) Absorption effectiveness of melt-mixed composites. (**d**) Absorption effectiveness of wetly-melt-mixed composites. (**e**) Total effectiveness of melt-mixed composites. (**f**) Total effectiveness of wetly-melt-mixed composites.

The electromagnetic interference shielding effectiveness is enhanced with the increasing number of the SWCNTs loading, as shown in Figure 5. The EMI shielding efficiency of the materials increases to 6.5 dB gradually with the number of SWCNTs via the melt-mixing process for poor dispersion, as shown in Figure 7a. However, the wetly-melt-mixed materials present a sharp increase when the loading phr is added from 0 to 2 phr before maintaining a certain level, as shown in Figure 7b. As shown in Figure 7c,d, the absorption effectiveness of melt-mixed composites and wetly-melt-mixed composites improves to a different level such that the maximum value of the latter is 17 dB and ten times that of the former. Meanwhile, the total effectiveness of wetly-melt-mixed composites is enhanced to 23.8 dB at the loading phr of 8 SWCNTs, which is almost three times that of the melt-mixed one, shown in Figure 7e,f.

As these composites are designed for multi-absorbing applications, the comparable examples are less effective. Nina [5] studied the EMI performance of butyl rubber-single walled carbon nanotube composites prepared via a solution-mixing process, and the shielding effectiveness was about 9~11.5 dB for the composite with 8 phr of SWCNT loading. Via water-induced modification and wetly-melt mixing, the EMI is 23.8 dB for composites prepared in our work, twice that in the mentioned research, indicating the effectiveness of the preparation method.

**Figure 8:**
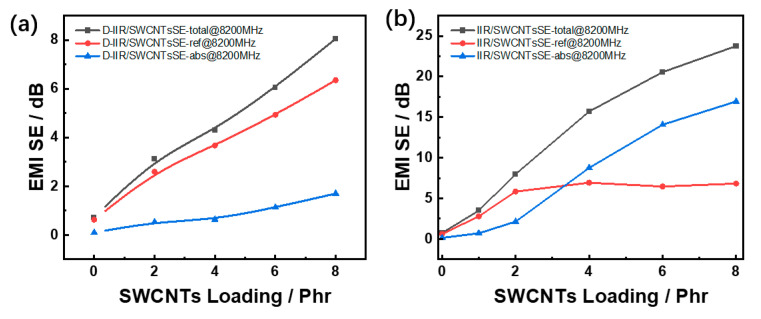
Electromagnetic interference shielding effectiveness of IIR/SWCNT composites with the loading phr of single-walled carbon nanotubes at the frequency of 8200 MHz. (**a**) Melt-mixed composites. (**b**) Wetly-melt-mixed composites.

From Figure 8a,b, the shielding effectiveness at the frequency of 8200 MHz of composites is related to the loading phr increase of SWCNTs via both melt-mixing and wetly-melt-mixing processes. Obviously, the wetly-melt-mixing process benefits the material in forming a good dispersive state to enhance the EMI shielding total effectiveness, and the dominant contribution factor of EMI performance of the materials is the absorption effectiveness.

### 3.7. Electromagnetic-Wave-Absorbing Properties

**Figure 9:**
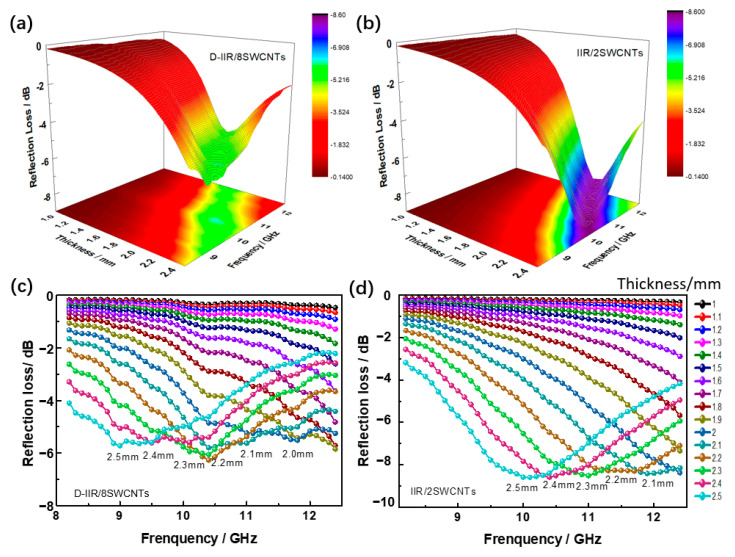
Electromagnetic-wave-absorbing properties of isobutylene isoprene rubber/SWCNT composites. (**a**) Three-dimensional image of reflection loss corresponding to thickness of the melt-mixed composite with 8 phr SWCNTs and incident wave frequency. (**b**) Three-dimensional image of reflection loss corresponding to thickness of the wetly-melt-mixed composite with 2 phr SWCNTs and incident wave frequency. (**c**) Reflection loss corresponding to thickness of the melt-mixed composite with 8 phr SWCNTs. (**d**) Reflection loss corresponding to thickness of the wetly-melt-mixed composite with 2 phr SWCNTs.

The three-dimensional image of reflection loss corresponding to thickness of melt-mixed composites and incident EM wave frequency presents an optimum value of −6.3 dB at 8 phr SWCNTs, as shown in Figure 9a. In Figure 9b, the optimum reflection loss of the wetly-melt-mixed composite is −8.7 dB with 2 phr SWCNTs. From Figure 9c, the best matching thickness of melt-mixed composite is 2.2 mm. For IIR/2SWCNTs, the matching thickness is 2.5 mm (see Figure 9d).

The water-induced modification of SWCNTs makes the wetly-melt-mixed composite construct a perfect net structure for the maximum wave absorbing performance with a lower dosage of SWCNTs.

### 3.8. Damping Properties

**Figure 10:**
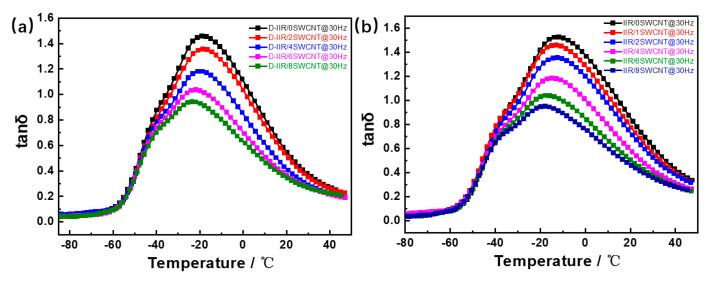
Effect of the amount of SWCNTs on the damping properties of IIR/SWCNT composites at the frequency of 30 Hz. (**a**) Melt-mixed composites. (**b**) Wetly-melt-mixed composites.

Butyl rubber composite material is an excellent option for damping applications. DMA tests of the composites were conducted at the frequency of 30 Hz in order to determine whether their damping properties were maintained and ascertain the effect of water-induced modification of SWCNTs on the damping properties as well as the effect of loading phr of SWCNTs (shown in Figure 10). As shown in Figure 10a, the main peaks appear at −18 °C and −40 °C in the curve of the damping factor. The peak temperature stays the same, and the value of the damping factor lowers as more single-walled carbon nanotubes are used.

The temperature ranges with damping factors over 0.2 of the wetly-melt-mixed composites are not as wide as that of the wetly-melt-mixed composites due to the uniform dispersion forming a continuous network of SWCNTs and rubber, as shown in Figure 10b. In shear mode, the response speed of the entanglement network of single-walled carbon nanotubes to strain is higher than that of rubber molecular segments, and the hysteresis loss of composites decreases.

## 4. Conclusions

The IIR/SWCNT composites produced via melt-mixing could not achieve good EMI performance with fewer CNTs. The tortuous configuration of poly-oxyethylene octyl phenol ether was induced with water with the aid of ultrasonication in OP-10 solution. Under the water-induced modification of single-walled carbon nanotubes, OP-10 could be wrapped with hydrophobic non-polar groups inside with hydrophilic groups outside to link water and SWCNTs with hydrogen bonds and further improve compatibility in the wetly-melt-mixing process. Electromagnetic interference shielding effectiveness of butyl rubber/single-walled carbon nanotube composites presents a huge enhancement due to good dispersion compatibility improvement with the aid of water-induced modification of SWCNTs. The SWCNTs are dispersed uniformly to form a complete network with a lower percolation threshold of wave-absorbing properties to 2 phr from 8 phr. With 8 phr SWCNTs, the tensile strength of the material was improved significantly from 7.1 MPa to 15.1 Mpa, and total electromagnetic interference shielding effectiveness of material was enhanced to 23.8 dB, three times that of the melt-mixed one. This demonstrates that the water-induced modification achieved good dispersion of SWCNTs for EMI shielding enhancement while keeping a wide damping temperature range from −55 °C to 40 °C with the damping factor over 0.2. IIR/SWCNT composite materials obtained in this work show great potential in the field of high damping EMI material application or multi-absorbing materials including electromagnetic wave absorbing and shocking absorbing service conditions in order to mitigate the vibration and EMI for sophisticated devices. The water-induced modification of CNTs could be applied to prepare other nonpolar rubber composites for applications in the field of electrostatic resistance, EMI, EM wave absorption, and flexible sensors.

## Data Availability

Not applicable.
